# Supplementary material for: Reconciling Mining with the Conservation of Cave Biodiversity: A Quantitative Baseline to Help Establish Conservation Priorities
Source: PLoS One. 2016 Dec 20;11(12):e0168348. doi: 10.1371/journal.pone.0168348 (PMC5173368; doi:10.1371/journal.pone.0168348)
Supplement: S1 Dataset — (ZIP) [file pone.0168348.s002.zip › Taxa/Serra Norte/SN_2013/N4WS/Lista N4WS-63.pdf]

CAVIDADE N4WS-0063

Classe	Ordem	Fam/Outros	Gên/Outros	Espécie	S-U	U-S	Zon.
Annelida	Oligochaeta			sp.	1		E
Arachnida	Acari	(Oribatida)		sp.1	X		E
Arachnida	Acari	Anystidae	*Erythracarus*	*nasutus*	X	X	E
Arachnida	Acari	Cosmochtoniidae		sp.1	X		E
Arachnida	Amblypygi	Phrynidae	*Heterophrynus*	*longicornis*	3		E
Arachnida	Araneae	Araneidae		jovem	X	X	E
Arachnida	Araneae	Corinnidae		jovem		1	E
Arachnida	Araneae	Drymusidae		jovem	X		E
Arachnida	Araneae	Ochyroceratidae	*Speocera*	sp.1		X	E
Arachnida	Araneae	Pholcidae	*Mesabolivar*	*aurantiacus*	X	X	E
Arachnida	Araneae	Pisauridae	*Thaumasia*	*velox*	2	3	E
Arachnida	Araneae	Salticidae		jovem	X		E
Arachnida	Araneae	Scytodidae	*Scytodes*	*globula*	2	2	E
Arachnida	Araneae	Tetragnathidae	*Azilia*	*histrio*		X	E
Arachnida	Araneae	Theridiidae	*Episinus*	sp.2	X	X	E
Arachnida	Araneae	Theridiosomatidae	*Plato*	sp.1	X	X	E
Arachnida	Opiliones	Sclerosomatidae	*Prionostema*	sp.1	X		E
Arachnida	Opiliones	Sclerosomatidae		jovem	X	X	E
Arachnida	Pseudoscorpiones	Chernetidae	*Spelaeochernes*	sp.1	X	X	E
Arachnida	Pseudoscorpiones	Chthoniidae	*Pseudochthonius*	sp.1		X	E
Entognatha	Collembola	Paronellidae		sp.1	X	X	E
Entognatha	Diplura	Campodeidae		sp.1		X	E
Entognatha	Diplura	Japygidae		sp.1	X		E
Insecta	Blattodea	Blattellidae		jovem	1		E
Insecta	Blattodea	Polyphagidae		jovem		1	E
Insecta	Coleoptera	Carabidae		sp.4	X		E
Insecta	Coleoptera	Curculionidae	Scolytinae	sp.2	X		E
Insecta	Coleoptera	Scydmaenidae		sp.2	X		E
Insecta	Coleoptera	Staphylinidae		sp.70		X	E
Insecta	Coleoptera	Staphylinidae		sp.73	X		E
Insecta	Coleoptera	Staphylinidae		sp.74	X		E
Insecta	Diptera	Agromyzidae		sp.		X	E
Insecta	Diptera	Ceratopogonidae		jovem		X	E
Insecta	Diptera	Chironomidae		sp.	X		E
Insecta	Diptera	Chloropidae		sp.	X		E
Insecta	Diptera	Limoniidae		sp.	X		E
Insecta	Diptera	Muscidae		jovem		X	E
Insecta	Diptera	Phoridae	*Trophodeinus*	sp.	X		E
Insecta	Diptera	Psychodidae	*Philosepedon*	sp.	X		E
Insecta	Diptera	Sciaridae	*Bradysia*	sp.	X		E
Insecta	Diptera	Streblidae	*Trichobius*	sp.	X		E
Insecta	Diptera	Tipulidae	Tipulinae	sp.	X	X	E
Insecta	Ephemeroptera	Leptophlebiidae		jovem	1		E
Insecta	Heteroptera	Cydnidae	*Pangaeus*	sp.1	X	X	E
Insecta	Homoptera	Cixiidae		jovem		X	E
Insecta	Hymenoptera	Apoidea		sp.1		1	E
Insecta	Hymenoptera	Formicidae	*Camponotus*	sp.1	X	X	E
Insecta	Hymenoptera	Formicidae	*Crematogaster*	sp.1	X		E
Insecta	Hymenoptera	Formicidae	*Crematogaster*	sp.3		X	E

Insecta	Hymenoptera	Formicidae	*Pachycondyla*	sp.1	X		E
Insecta	Hymenoptera	Formicidae	*Pachycondyla*	*striata*	1	1	E
Insecta	Hymenoptera	Formicidae	*Pheidole*	sp.2	X		E
Insecta	Isoptera	Termitidae	*Nasutitermes*	sp.	X	X	E
Insecta	Lepidoptera	Noctuoidea		sp.1	1	1	E
Insecta	Neuroptera	Myrmeleontidae		jovem		1	E
Insecta	Orthoptera	Phalangopsidae	*Paraclodes*	sp.		1	E
Insecta	Orthoptera	Phalangopsidae	*Phalangopsis*	sp.	32	3	E
Insecta	Thysanura	Nicoletiidae		sp.1	X		E
Malacostraca	Isopoda	Armadillidae		sp.1	X		E
Malacostraca	Isopoda	Philosciidae		sp.1	X		E
Malacostraca	Isopoda	Philosciidae		sp.2		X	E
Amphibia	Anura	Dendrobatidae	*Epipedobates*	sp.	1		E
Amphibia	Anura	Leptodactylidae	*Leptodactylus*	cf. *vastus*		1	E
Amphibia	Anura	Strabomantidae	*Pristimantis*	cf. *fenestratus*	4	1	E
Mammalia	Chiroptera	Furipteridae	*Furipterus*	*horrens*		2	E
Mammalia	Chiroptera	Phyllostomidae	*Carollia*	*perspicillata*	40	1	E
Reptilia	Squamata			sp.3	1		E
